# Some Factors in the Pathology of Gas Gangrene

**Published:** 1917-12

**Authors:** 


***Some Factors in the Pathology of Gas Gangrene.*** By W. d'Este Emery, Capt. R. A. M. C. Abs. from the *Lancet*, May 6, 1916.

E. believes that B. aerogenes capsulatus is the only organism that can be considered the actual cause of the disease. He notes the low pathogenicity of this bacillus under ordinary conditions as contrasted with its excessive virulence in the phenomena of gas gangrene, and seeks an explanation chiefly in leucocytic activity. He finds that ordinarily B. aerogenes capsulatus is killed by blood-serum and blood-plasma, but that it is much more a prey to leucocytes, which under ordinary circumstances, form a sufficient barrier to its growth. By means of an emigration chamber he has shown *in vitro* that a weak solution of the toxin of the bacillus has a positive chemotactic action on leucocytes. These observations sufficiently explain, E. believes, the fact that most cases, in which the organisms are present, do not succumb to infection.

He then asks why in a limited number of cases infection of a serious nature takes place. He thinks it due, in the first place, to the inhibition of leucocytosis by a weakening of the blood supply in any way whatever. He cannot, however, agree with Taylor that the mechanical action of the gas generated by the organism plays any part, for, he declares, " gangrene may occur, and the patient may die, without the formation of any gas in the tissues, and in many cases of severe disease there is but little, and none in the advancing edge. " He does not agree with Taylor that the infection is necessarily one of the muscles, but thinks its frequency in muscle tissue is due merely to the larger sugar supply that materially increases the gas production of the organism.

The chief agency, however, in breaking down the defence he believes to be the toxin of the bacillus which " inhibits emigration and kills the leucocytes when present in large amount. " From experiments *in vitro* he concludes : " If toxin is formed in sufficient amount the defence of the body, so far as it is entrusted to the leucocytes, breaks down altogether; the leucocytes are not attracted to the infected region, and those that are there already, all too scanty as they are, are killed. "

He next considers the conditions most likely to lead to a large production of toxin. He finds that the organism grows with great rapidity in favorable conditions, and he thinks the most favorable to be the presence of dead tissues and especially " dead blood ". He thinks hemorrages are doubly dangerous because they not only supply favorable foci themselves, but cause swelling that may shut off the blood supply. He notes that thrombosis occurs when the toxin reaches the veins, and he thinks that if the gas acts mechanically, as Taylor suggests, it does so only in occluding the veins.

As to treatment, he thinks that the only sure measures are : early drainage, removal of clot and dead tissues, and encouragment of the circulation. He notes, however, a very rapid destruction of the red blood cells and the condition of acidosis established by Wright. He thinks that an intravenous injection of 300 to 400 cc. of a 4 o/o solution of sodium bicarbonate tends to lessen the toxic symptoms.

